# Development and Validation of an E2F-Related Gene Signature to Predict Prognosis of Patients With Lung Squamous Cell Carcinoma

**DOI:** 10.3389/fonc.2021.756096

**Published:** 2021-10-22

**Authors:** Cailian Wang, Xuyu Gu, Xiuxiu Zhang, Min Zhou, Yan Chen

**Affiliations:** ^1^ Department of Oncology, Zhongda Hospital, School of Medicine, Southeast University, Nanjing, China; ^2^ School of Medicine, Southeast University, Nanjing, China

**Keywords:** LUSC, E2F pathway, gene signature, prognosis, risk score

## Abstract

**Background:**

Lung squamous cell carcinoma (LUSC) generally correlates with poor clinical prognoses due to the lack of available prognostic biomarkers. This study is designed to identify a potential biomarker significant for the prognosis and treatment of LUSC, so as to provide a scientific basis for clinical treatment decisions.

**Methods:**

Genomic changes in LUSC samples before and after radiation were firstly discussed to identify E2 factor (E2F) pathway of prognostic significance. A series of bioinformatics analyses and statistical methods were combined to construct a robust E2F-related prognostic gene signature. Furthermore, a decision tree and a nomogram were established according to the gene signature and multiple clinicopathological characteristics to improve risk stratification and quantify risk assessment for individual patients.

**Results:**

In our investigated cohorts, the E2F-related gene signature we identified was capable of predicting clinical outcomes and therapeutic responses in LUSC patients, besides, discriminative to identify high-risk patients. Survival analysis suggested that the gene signature was independently prognostic for adverse overall survival of LUSC patients. The decision tree identified the strong discriminative performance of the gene signature in risk stractification for overall survival while the nomogram demonstrated a high accuracy.

**Conclusion:**

The E2F-related gene signature may help distinguish high-risk patients so as to formulate personalized treatment strategy in LUSC patients.

## Introduction

Lung cancers remain the leading cause of cancer-related death worldwide (1). Nonsmall cell lung cancer (NSCLC) is the predominant subtype of lung cancers accounting for approximately 85%, of which more than 30% cases are lung squamous cell carcinomas (LUSC) (2). LUSC, as compared with lung adenocarcinoma (LUAD), correlates with more adverse clinical prognoses, and there is a lack of available targeted drugs. Radiotherapy and chemotherapy are traditional treatment strategies (3), while there is a high risk of treatment failure in patients with advanced LUSC due to the development of treatment resistance (4). Despite the fact that immunotherapy has shown great potential in treatment of LUSC over the past years, it brings benefits to a limited population (5). It was reported that the 5-year overall survival (OS) rate in patients with stage I/II LUSC was about 40%, and even lower to 5% when a stage III/IV LUSC was present (6). Currently, basic biomarkers and precise targets for the prognosis and treatment of LUSC are still unclear. In this setting, further research into the potential prognostic biomarkers of LUSC is required, so as to provide better prognostic prediction and individualized treatment. Similar to many other carcinomas, the initiation and progression of LUSC are closely related to the dysregulation of cell cycle (7, 8). The timing of the cell to proliferate, to enter reversible quiescent phase, to differentiate, or to apoptosis is controlled by the cell cycle clock apparatus (9). Dysregulation of the cell cycle process is a necessary step in malignant transformation (10).

The E2 factor (E2F) pathway is a major pathway involved in the cell cycle in mammals, and the E2F family of transcription factors play various biological roles including cell cycle control (11). Research found that the cell cycle-related E2F genes are significantly associated with the prognosis of lung cancer patients and provide a potential therapeutic strategy (12). Nevertheless, to our knowledge, there has been no study reporting the discriminative role of the E2F family in identifying high-risk LUSC. In this study, we explored the genomic changes in LUSC samples before and after radiatiotherapy to identify E2F pathway as the potential risk factor for prognosis in LUSC patients. An E2F-related prognostic gene signature was then established and further validated in additional independent cohorts. Finally, a decision tree and a nomogram were established according to the gene signature and multiple clinicopathological characteristics to improve risk stratification and quantify risk assessment for individual patients.

## Materials and Methods

### Data Processing

The microarray dataset GSE42172 which contained paired normal A549 lung cancer cells (*n* = 6) and radiation-exposed A549 cells (*n* = 6) was selected to explore the genomic changes before and after radiation. Also, the clinical annotations and follow-up information of 916 LUSC patients across different platforms were included in this study. The datasets GSE29013, GSE30219, and GSE37745 were downloaded from Affymetrix Human Genome U133 Plus 2.0 Array GPL570, and the expression data of these datasets were integrated using the R package combat (**Supplementary Figures S1A, B** **)** after eliminating batch effects. After integration, 166 patients in this cohort were enrolled in the training set. The datasets GSE14814, GSE17710, GSE42127, and GSE74777 from different platforms were used as a validation set 1 after integration using the combat package, which contained 266 patients. In addition, RNA-Seq data in FPKM of 499 patients who met the criteria were obtained from TCGA, and the expression data were taken as a validation set 2 after normalization by transcripts per kilobase per million (TPM).

### Signature Establishment

The gene set variation analysis (GSVA) was conducted to evaluate changes of cancer biomarkers obtained from the Molecular Signatures Database (MSigDB) before and after radiotherapy in the dataset GSE42172 (13). Markers of significant changes in the training set (*t* > 1) were quantified using single-sample gene set enrichment analysis (ssGSEA) (14). A univariate Cox proportional hazard (COX-PH) regression model was utilized to assess the prognostic value of diverse cancer biomarkers for LUSC patients. Multiscale embedded gene coexpression network analysis (MEGENA) (15), an R package with performance superior to coexpression network analysis, was performed to analyze the genes with standard deviation >0.9, and the planar filtered network (PFN) was plotted based on the gene expression correlation. A LUSC-specific gene network composed of interconnected subnetworks or modules was constructed using the multiscale clustering method, and the module feature genes were identified using moduleEigengenes R function to calculate the correlation between the modules and the E2F signaling pathway and to determine the most relevant module. With the *p*-value in COX-PH <0.05 as the threshold, 53 candidate genes from the E2F-related module were screened out. Then, a least absolute shrinkage and selection operator (LASSO) regression model was employed to further screen reliable prognostic indicators (16). The standardized gene expression values weighted using corresponding LASSO coefficients were included, and a risk score related to the E2F signaling pathway, E2F-related score (ERS), was established as follows:


$$\text{ERS}=\Sigma \text{i Coefficient }(\text{mRN}{\text{A}}_{\text{i}})\times \text{Expression }(\text{mRN}{\text{A}}_{\text{i}}).$$

### Bioinformatics and Statistics

GSEA was implemented to verify the E2F signaling pathway enrichment in the high-ERS group with the E2F-target genome from MSigDB (17). Date analysis and graph plotting were carried out using R software (version 4.0.4, http://www.r-project.org). The survival analysis was completed with the Kaplan-Meier method along with log-rank test. Additionally, the prognostic value of each parameter for OS was evaluated using a COX-PH model. A time-dependent receiver operating characteristic (tROC) curve was drawn to assess the predictive value of ERS assisted by the R package “survivalROC,” followed by comparison of the areas under the curve at different time points (AUC(t)). Meta-analysis (*I* ^2^  <30%, fixed model) was carried out to assess the prognostic significance in the merged cohort. Afterwards, consensus clustering of patients was conducted using the R package “ConsensusClusterPlus” based on the expression of candidate genes, whereby evaluating the discriminative performance of candidate genes (18). A decision-making tree was created for risk stratification with recursive partitioning analysis (RPA) utilizing the R package rpart (19). Two independent datasets, IMvigor210 and a dataset containing 47 responders with melanoma to immunotherapy, were downloaded and analyzed (20). The IMvigor210 dataset was derived from the freely available, fully documented software and data package under the Creative Commons Attribution 3.0 license from http://research-pub.gene.com/IMvigor210CoreBiologies. A sum of 298 patients with urothelial carcinoma who had complete clinical data and 47 patients with skin melanoma who had underwent immunotherapy were integrated to identify the value of ERS for immunotherapy. The Tumor Immune Dysfunction and Exclusion (TIDE) algorithm was utilized to evaluate the value of ERS in clinical immunotherapy. The R package “rms” was utilized to draw nomogram and calibration curve (21). Decision curve analysis (DCA) was carried out by Wilcox test with the DCA package to test the difference between two groups (22). Differences among multiple groups were examined by the Kruskal-Wallis test and the differences among categorical data were processed by the Chi-square test.

## Results

### Workflow of the Study

**Figure 1:**
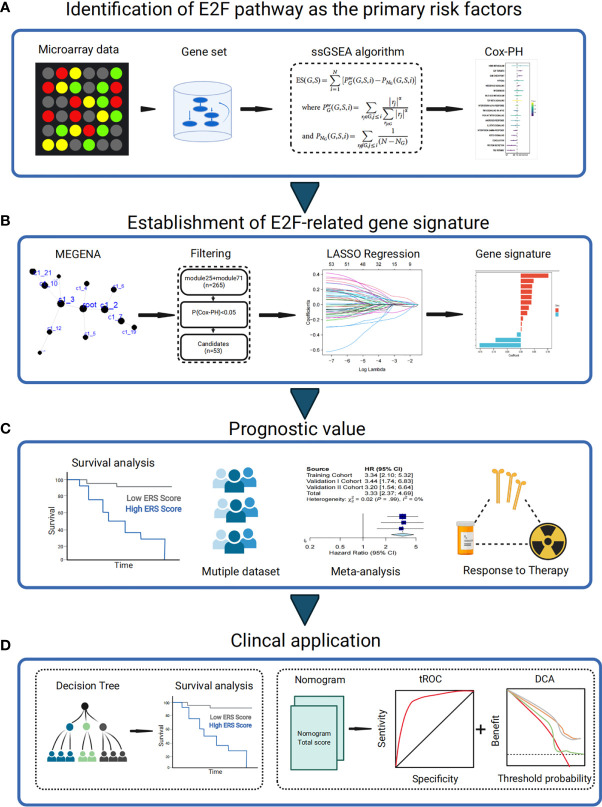
Schematic diagram of the study design. **(A)** E2F signaling pathway was identified as the main risk factor for the prognosis of LUSC patients. **(B)** Stable E2F-related gene signature for predicting prognosis was generated using combined methods. **(C)** The prognostic value of gene signature was validated in different cohorts. **(D)** Clinical application. Cox-PH, Cox proportional hazards; LASSO, least absolute shrinkage and selection operator; LUAD, lung adenocarcinoma; ssGSEA, single-sample gene set enrichment analysis; tROC, time-dependent receiver operating characteristic; MEGENA, weighted gene coexpression network analysis.

First, E2F was one of the significantly changed pathways after radiation. The E2F signaling pathway was demonstrated as the main risk factor for the prognosis of LUSC patients (**Figure 1A**). Then, MEGENA, univariate COX-PH, and LASSO analyses were conjunctively employed to filter candidates and to construct an E2F-related gene signature of survival significance (**Figure 1B**), which was further assessed using the training and two external validation sets. Additionally, its prognostic capability was verified and the response to treatment was evaluated by meta-analysis to determine its potential as a promising prognostic marker (**Figure 1C**). At last, a decision tree was established to improve risk stratification, along with a nomogram generated to quantify the risk evaluation and survival probability of individuals on the basis of ERS and multiple clinicopathological characteristics (**Figure 1D**).

### The E2F Signaling Pathway Is a Major Risk Factor for Radiotherapy Response in LUSC

**Figure 2:**
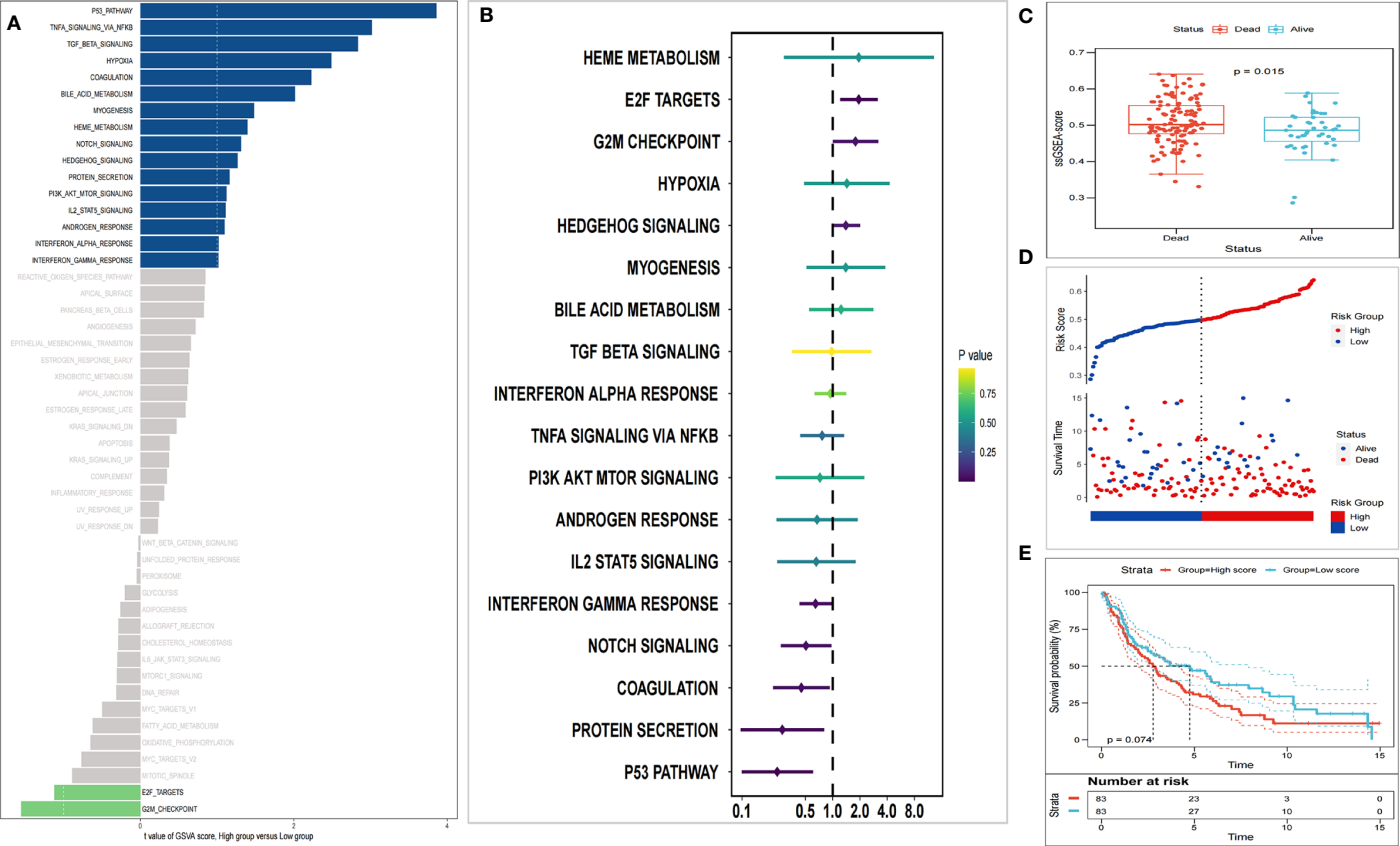
E2F target is identified as the main risk factor of survival after radiation. **(A)** GSVA analysis showed significant changes in 18 cancer-related pathways (*t* > 1). **(B)** Univariate Cox regression analysis exhibits that E2F targets were the main risk factors among diverse cancer biomarkers. **(C)** The E2F ssGSEA score of patients who died during the follow-up period increased significantly. **(D)** Kaplan-Meier analysis suggested poorer OS of patients with higher E2F ssGSEA scores. **(E)** Patients with higher E2F scores have shorter survival.

The analyzed results of the radiation dataset in GSE42172 showed that 18 cancer-related pathways were markedly changed after radiation (*t* > 1), in which two pathways including the E2F signaling pathway were notably downregulated, and 16 pathways including p53 signaling were notably upregulated (**Figure 2A**). According to the ssGSEA score of the 18 changed pathways and the OS data in the training set, each pathway was conferred a Cox coefficient. Accordingly, the E2F signaling pathway exerted a greater effect on survival than other cancer-related pathways (such as cell cycle, signal transduction pathway, EMT, angiogenesis, apoptosis, etc.) (**Figure 2B**). During the follow-up period, remarkable higher E2F ssGSEA scores were observed in the dead patients as compared with the surviving patients (**Figure 2C**). In the training set, two groups were divided according to the median E2F ssGSEA score. The results showed a lower OS rate (**Figure 2D**) and shorter average survival time (**Figure 2E**) in the high-score group.

### Establishment of E2F-Associated Prognostic Gene Signature

**Figure 3:**
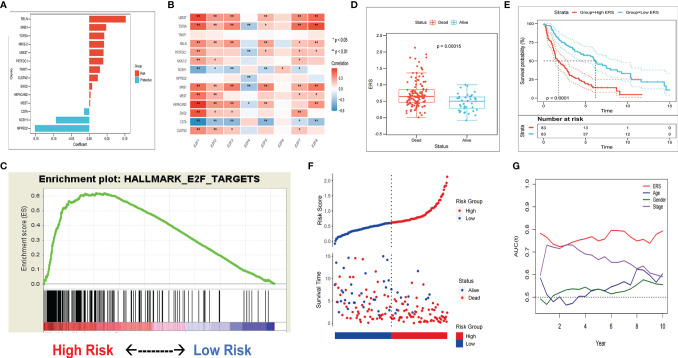
The gene signature predicts worse survival of patients in the training set. **(A)** Distribution of LASSO coefficients of the E2F-related gene signature. **(B)** Association between gene signature and the E2F transcription factor family. **(C)** GSEA validated E2F pathway enrichment in the high-ERS group. **(D)** ERS was significantly increased in patients who died during follow-up. **(E)** The patients in the high-ERS group had worse survival. **(F)** Kaplan-Meier analysis showed a worse OS of patients with higher ERS. **(G)** tROC analysis showed ERS to be an accurate variable for survival prediction.

In the training set, MEGENA analysis was conducted with whole-transcriptome profiling data and E2F ssGSEA score. We observed a minimum error rate of the model when scale = 7 (**Supplementary Figures S2A–D**). A LUSC-specific gene network with 70 modules was generated (**Supplementary Figure S3A**). Among these modules, module 25 and its submodule 71 shared the closest association with E2F ssGSEA score (*r* = 0.52, *p* = 5e−13/*r* = 0.53, *p* = 2e−13) (**Supplementary Figures S3B** and **S4A**). The genes extracted from modules 25 and 71 were subjected to univariate COX-PH analysis, and 53 promising candidate factors (47 risk factors and six protective factors) were identified with the threshold of *p* < 0.05 (**Supplementary Figure S3C**). Next, the LASSO regression model was utilized to determine the most reliable prognostic factors. Using a 10-fold cross-validation to avoid overfitting, the optimal *λ* value 0.06779023 was selected (**Supplementary Figures S3D** and **S4B**). The remaining 11 genes had their own nonzero coefficients (**Figure 3A**). Finally, ERS was calculated according to the formula:


$$\text{ERS}=\Sigma \text{i Coefficient }(\text{mRN}{\text{A}}_{\text{i}})\times \text{Expression }(\text{mRN}{\text{A}}_{\text{i}}).$$

### ERS Is a Risk Factor for OS in Each Set

**Figure 4:**
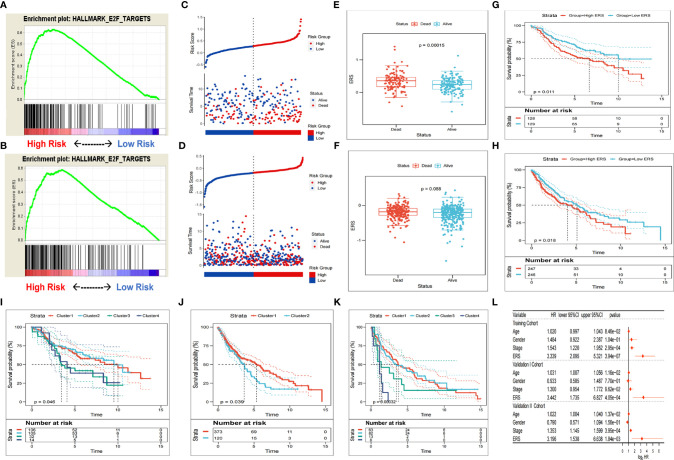
Verification of gene signatures in different sets. **(A, B)** GSEA validated E2F pathway enrichment in the validation I and II sets. **(C, D)** The dead patients in the validation I and II sets showed higher ERS. **(E, F)** The patients of the high-ERS group in the validation I and II sets had worse survival. **(G, H)** Patients with higher ERS have a poorer prognosis in the validation I and II sets. **(I–K)** On the basis of the expression patterns of gene signature, the survival rate of clusters derived from consensus clustering differed greatly. **(L)** Multivariate Cox regression analysis showed ERS as an independent risk factor for OS in the validation I and II sets.

In the training set, most risk factors exerted positive correlations with E2F transcription factor (**Figure 3B**). With the E2F target genome from MSigDB, GSEA results demonstrated more abundant enrichment of the E2F signaling pathway in the high-ERS group (**Figure 3C**). The patients who died during the follow-up period exhibited notably higher ERS compared with the surviving patients (**Figure 3D**), and the patients in the high-ERS group showed markedly poorer survival (**Figure 3E**). Results of Kaplan-Meier analysis exhibited worse prognoses of patients with higher ERS scores *versus* those with lower scores (**Figure 3F**). Among a variety of clinicopathological variables, the multivariate COX-PH model identified the American Joint Committee on Cancer (AJCC) TNM staging and ERS as two independent risk factors for OS in the training set. In addition, tROC analysis demonstrated ERS as the most accurate predictive biomarker for OS (**Figure 3G**). Furthermore, the patients were assigned into two groups by consensus clustering with the optimal *k* value as the threshold, which showed remarkably different prognoses, indicative of the good potential of the ERS to distinguish patients with different prognostic risks (**Figure 4I** and **Supplementary Figure S5A**).

To validate the prognostic robustness of E2F-associated gene signature in diverse sets, two external sets were selected for validation. Similarly, in the validation sets 1 and 2, more E2F signaling pathway enrichment was verified in the high-ERS group with the E2F target genome set by GSEA (**Figures 4A, B** **)**. The dead patients had a noticeable higher ERS than the surviving patients in cohort 1, yet no significant variance was noted in cohort 2 (**Figures 4C, D** **)**. The patients with high scores had markedly poorer survival (**Figures 4E, F** **)**. The results of the Kaplan-Meier analysis further revealed that the OS rate predicted by high ERS was lower than that predicted by low ERS (**Figures 4G, H** **)**. The cohort was grouped into different subtypes with consensus clustering with the optimal *k* value as the threshold, and the prognosis differed between subtypes (**Figures 4J, K** and **Supplementary Figures S5B, C**). In addition, multivariate COX-PH analysis suggested ERS be independently prognostic for adverse OS (**Figure 4L**).

### ERS Indicates Poor Survival in the Pooled Cohort and Can Be a Potential Biomarker for Therapeutic Resistance

**Figure 5:**
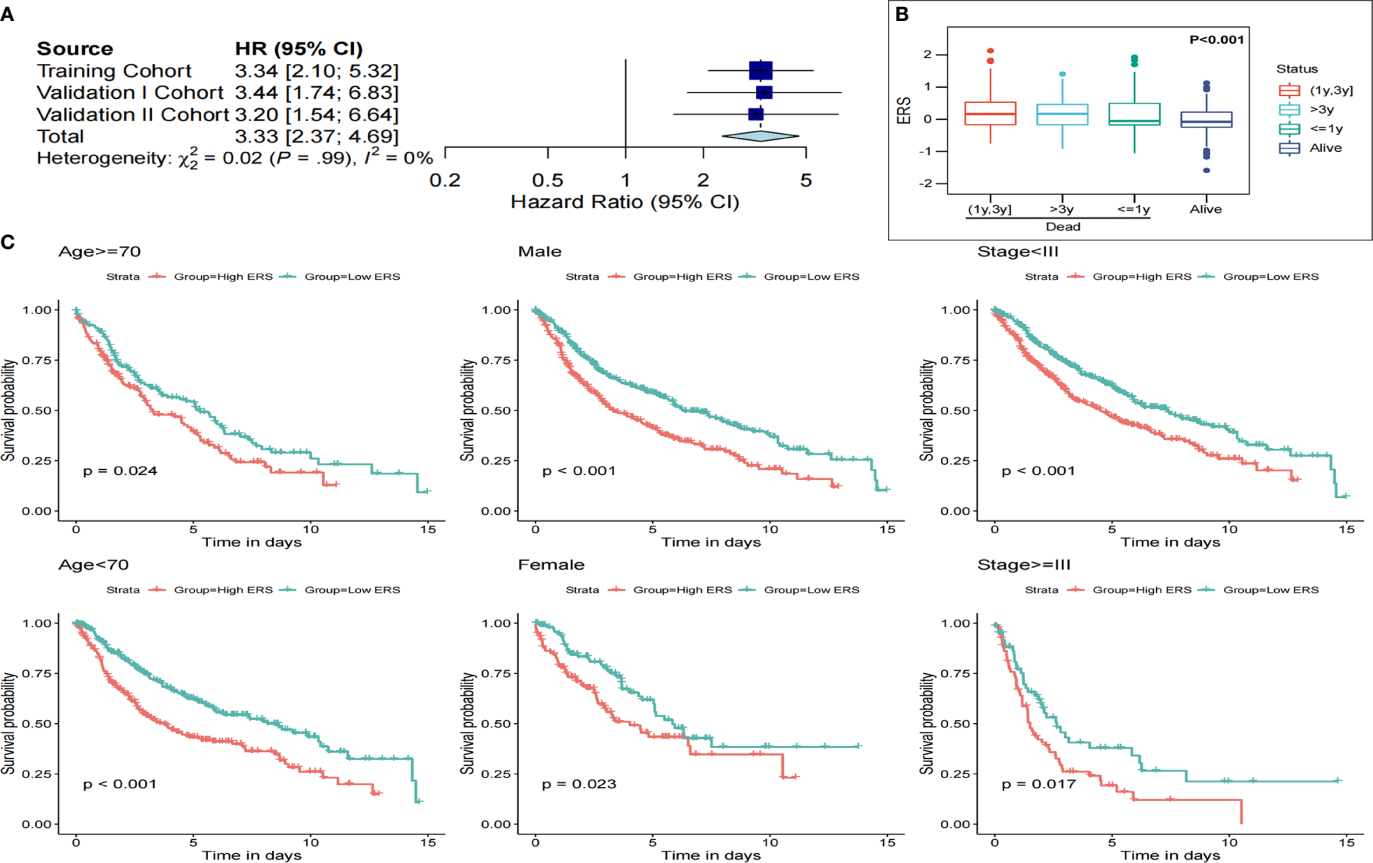
ERS is a valuable indicator of poor survival in the pooled cohorts and subgroups. **(A)** Meta-analysis results showed ERS to be a valuable prognostic marker. **(B)** The ERS score was markedly raised in the dead patients, especially in those who had a shorter survival. **(C)** ERS distinguished high-risk patients in different subgroups, including age, gender, and AJCC staging.

Meta-analysis was conducted to assess the prognostic significance of E2F-related gene signature in the pooled cohort of one training set and two verification sets. Consequently, patients with high ERS showed worse prognoses than patients with low ERS (**Figure 5A**). In total, 916 patients from the three sets were integrated for further investigation. The ERS was upregulated significantly in deaths at follow-up, even higher in those with a shorter survival time (**Figure 5B**). ERS could also distinguish the high-risk patients suffering from adverse outcomes from different subgroups, such as different clinicopathological characteristics, including gender, age, and TNM stage (**Figure 5C**).

**Figure 6:**
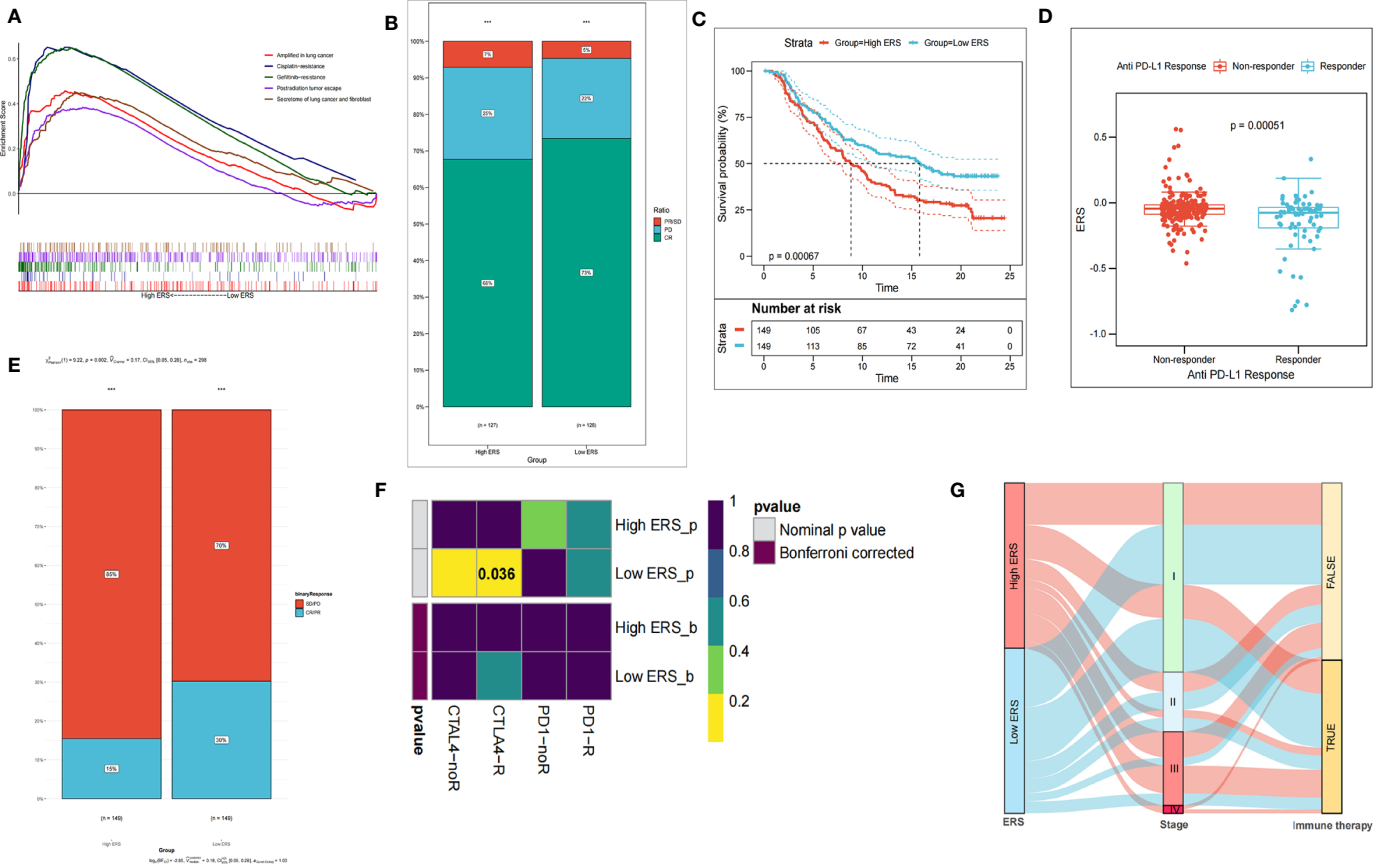
ERS gene signature is a promising biomarker of resistance to different treatments. **(A)** GSEA predicts the correlation of gene signature with resistance to chemotherapy and resistance to radiotherapy. **(B)** The proportion of adverse postoperative outcomes increased in the high-ERS group. **(C)** In the IMvigor210 cohort, the prognosis of patients with higher ERS was remarkably worse. **(D)** ERS scores of groups with different anti-PD-1 clinical responses. **(E)** In the IMvigor210 cohort, the patients with higher ERS to anti-PD-L1 immunotherapy exhibited a lower clinical response rate. **(F)** Heat map displayed response to immunotherapy. **(G)** Sankey diagram showed the immunotherapy response predicted by the TIDE algorithm. ***P < 00.01.

Considering that the E2F signaling pathway may enhance the resistance to treatment, we probed into whether ERS is a biomarker of therapeutic resistance. It was predicted by GSEA that higher ERS was strikingly correlated with resistance to diverse treatments (such as chemotherapy, radiotherapy, and targeted therapies) (**Figure 6A**). Subsequently, therapeutic information and clinical outcome were downloaded from TCGA to verify the prediction. Following primary surgical treatment, compared with the low ERS group, the ratio of patients in the high-ERS group with the progressive disease to that of patients with partial remission or stable disease was prominently upregulated (**Figure 6B**).

Subsequently, we assessed the value of the ERS in predicting the therapeutic outcomes of patients. To this end, patients with anti-PD-L1 immunotherapy in the IMvigor210 cohort were assigned into high ICI score and low ICI score subgroups. It was worthy to note that in the IMvigor210 cohort, the patients with low ERS had significantly longer survival time than those with high ERS (**Figure 6C**). Besides, the lower ERS was associated with the objective response to anti-PD-L1 treatment (**Figure 6D**), and the objective response rate of anti-PD-L1 treatment was higher in the low-ERS group than that in the high-ERS group (**Figure 6E**). The Submap module in the GenePattern was utilized for evaluation and comparison of the patients in the training set and 47 responders to immunotherapy. As compared with the high-score group, anti-CTL4-A treatment was more effective for the low-score group (*p* = 0.036) (**Figure 6F**). With the response to immunotherapy predicted by the TIDE algorithm, the low-score group was more likely to respond to immunotherapy, while there was no evident difference between the two groups (**Figure 6G**) (Chi-square test, *p* > 0.05).

### The Combination of ERS and Clinicopathological Characteristics Contributes to Improving Risk Stratification and Survival Prediction

**Figure 7:**
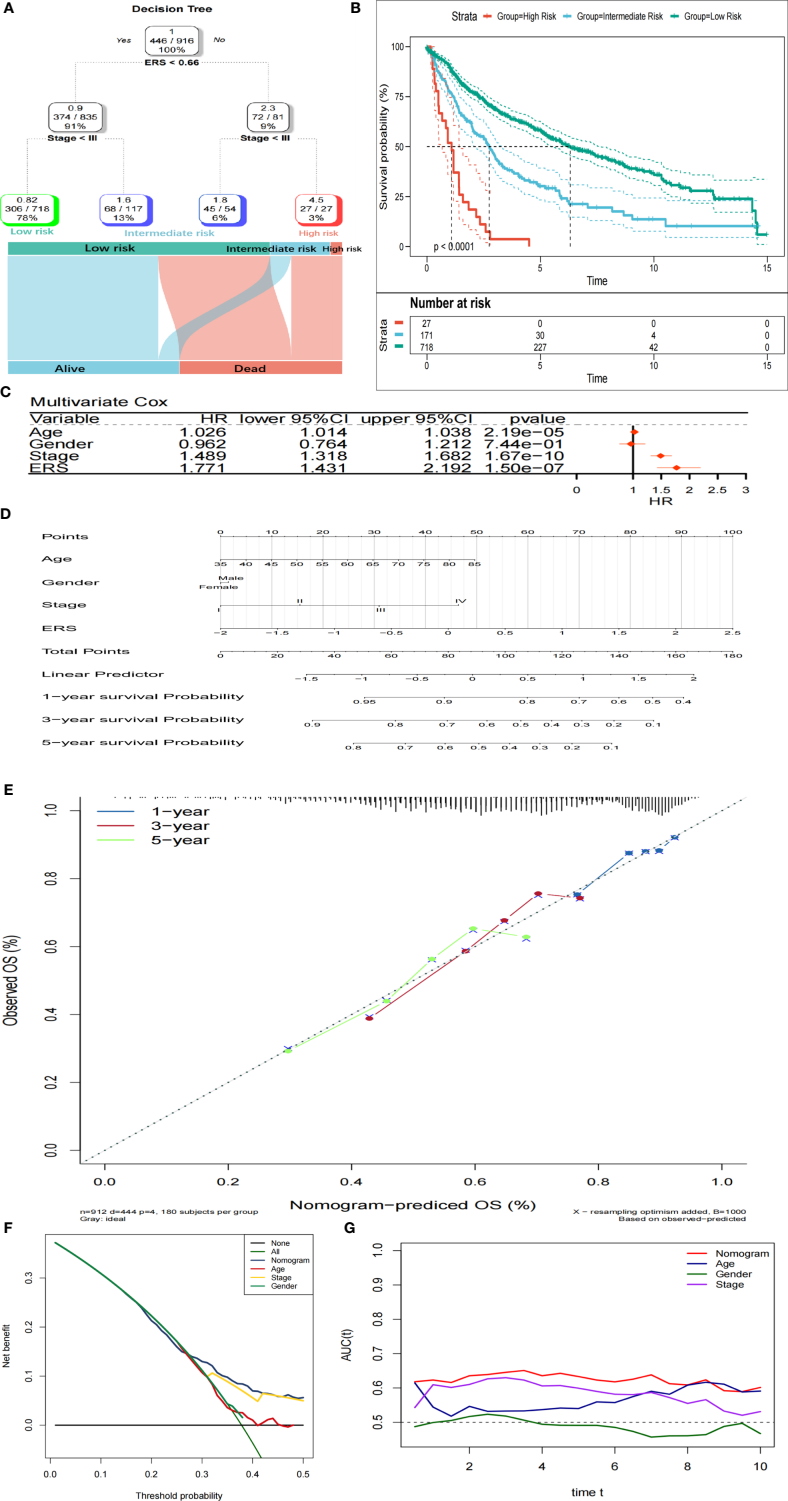
Combination of ERS and clinicopathological characteristics contributes to better risk stratification and survival prediction. **(A)** Risk stratification was improved by constructing a decision tree. **(B)** Kaplan-Meier analysis showed noticeably different prognoses of different risk strata. **(C)** In the whole cohort, ERS was the most important risk factor for OS. **(D)** Risk assessment of individuals was quantified by constructing a nomogram. **(E)** Calibration analysis revealed high accuracy of survival prediction. **(F)** DCA analysis indicated that nomogram has the optimum decision benefit under most thresholds. **(G)** tROC analysis demonstrated that the nomogram was the most stable and powerful indicator for OS among all the clinical variables.

Four parameters were available for 916 LUSC patients, namely age, gender (male or female), TNM stage, and ERS. After risk stratification using the decision tree, only the TNM stage and ERS remained in the decision tree, and three different risk subgroups were identified (**Figure 7A**). It was noteworthy that the ERS was the optimum stratification factor. The OS rates showed noticeable differences among these three risk subgroups (**Figure 7B**). Multivariate COX-PH analysis results indicated ERS to be the optimum prognostic indicator (**Figure 7C**). In order to quantify the risk assessment and survival probability of LUSC patients, a nomogram was generated using ERS and other clinicopathological characteristics (**Figure 7D**). According to the calibration analysis, the 1-, 3-, and 5-year survival probability predicted by the nomogram nearly reached the ideal results (**Figure 7E**), indicating high accuracy of the nomogram. Furthermore, the 3-year DCA revealed that the nomogram had optimum decision benefit at most thresholds (**Figure 7F**). In comparison with other characteristics, the nomogram exerted the most powerful and stable capability for predicting survival, with an average area under the curve above 0.6, considerably superior to the pathological TNM staging (**Figure 7G**).

## Discussion

Surgery is the main treatment strategy of NSCLC, with chemoradiotherapy, targeted therapy, and immunotherapy as adjuvants (23). However, it was estimated that more than 85% of patients with NSCLC have lost optimum time for surgical treatment at the first diagnosis, and only 25% to 30% can be treated by the traditional surgical resection (24). With the continuous development of computer technology, radiobiology, and functional imaging in recent years, radiotherapy has shown considerable advantages in the treatment of patients with locally advanced NSCLC (25). Existing research unraveled that radiotherapy is safe and effective for patients with stage I NSCLC, hence, radiotherapy is the primary choice for patients with early lung cancer who are elder or have poor cardiopulmonary function, rather than surgery (26). Due to the demands for precision medicine, the importance of radiotherapy has been highlighted, but the sensitivity to radiotherapy is a limiting factor for its therapeutic effect (27). Besides, few reports are focusing on the changes in the pathways before and after radiotherapy for NSCLC. Identification of biomarkers to estimate the prognosis of patients electing to receive radiotherapy is of importance in the clinical management of NSCLC (28). The E2F transcription factor family plays a crucial role in regulating cell cycle progression, while the E2F-RB1 pathway is dysregulated in approximately 90% of lung cancers (29). It was uncovered that enhanced E2F activity contributes to the activation of nAChR (encoded by CHRNA5) by its ligands (such as nicotine) in the neurons, whereby promoting radioresistance through facilitating cell cycle progression (30). Radiotherapy is commonly used in the clinical treatment of LUSC, so we aim at identifying whether the E2F signaling pathway can serve as a prognostic indicator of LUSC.

In this study, We first explored that the E2F pathway was identified as the mainly changed pathway after radiation using the “GSVA” algorithm in the GSE42172 dataset. We then used all of the changed pathways and the clinical data in the training set to apply Cox regression, and we find that the E2F pathway is the best prognostic factor. Therefore, we chose E2F pathway for subsequent analysis. MEGENA was performed to identify LUSC-specific E2F-related gene modules based on whole-transcriptome profiling data, and then Cox univariate and LASSO regression models were used to screen prognostic biomarkers, which were taken to establish an E2F-related gene signature of prognostic value. A risk scoring system based on the signature, called ERS here, was then constructed. Survival analysis identified that ERS was a risk factor for the OS of patients in each cohort, and a higher ERS was associated with a worse survival outcome. The prognostic value of the gene signature was further validated in two independent cohorts derived from different platforms. In the meta-analysis and subgroup analysis, ERS was still capable of discriminating high-risk patients, suggesting that the performance of ERS is reliable in pooled populations and similar-stage subgroups. In groups of adjuvant therapy, patients with higher ERS suffered from worse survival outcomes as compared with those with lower ERS. Patients with lower ERS gained more benefits from CTL4-A and PD-L1 treatments, which might be associated with the gene signature-derived resistance to therapies, indicating the potential role of the gene signature as a promising marker of therapeutic resistance in LUSC patients.

Moreover, a decision tree combining the ESR and multiple clinicopathological characteristics was constructed to improve risk stratification. We found that only the TNM stage and ERS remained in the decision tree, and three different risk subgroups were identified. Among the three subgroups, significant difference was noted regarding OS. The ERS was identified as the predominant discriminative factor, which was further validated by the multivariate COX-PH analysis. These collectively suggest that the E2F-related gene signature is potentially a powerful risk factor for OS of LUSC patients. In subsequent work, a nomogram was generated to quantify the risk assessment for individual patients, with the involvement of the ERS and other clinicopathological characteristics. On calibration curves, the predicted results appeared to highly approach to the actual outcomes, indicative of a high accuracy of the nomogram in prognosis prediction. In addition, tROC analysis demonstrated that the nomogram performed the best on survival prediction at different time points during follow-up, as compared with other variables.

Of the biomarkers involved in the gene signature, some have been studied in many cancers, while most of them are rarely investigated in LUSC. It is proven that E2F-related genes have great implications in cell cycle, proliferation, differentiation, and apoptosis, and they are regarded as the determinant of the timing for G1/S transition. An animal experiment demonstrated that the increased expression of E2F activators may result in upregulation of E2F target genes and a risk of spontaneous cancer formation. There have been studies reporting the dysregulated expression of E2F activators in multiple human malignancies, such as bladder, breast, ovarian, prostate, gastrointestinal, and lung cancers. Although high-level E2F activators and its associations with clinicopathological characteritics and prognosis have been partly reported in human NSCLC, to the best of our knowledge, its role in LUSC has not been probed. In this setting, we here developed a risk scoring system, ERS, to improve the prediction for the survival of LUSC patients, and further validated its performance in external independent cohorts, which outperformed conventional immunotherapeutic biomarkers.

The retrospective nature of our study is an inevitable limitation. Although we included as many datasets as possible for rigorous validation and combined multiple different approaches to reduce batch effects, sampling bias caused by tumor genetic heterogeneity and cross-platform integration could only be reduced but not completely eliminated. Meanwhile, further experimental studies are required to elucidate tumor E2F-related biological functions underlying the gene signature in LUSC.

## Conclusion

To sum up, a novel E2F-related gene signature was established here to discriminate high-risk LUSC patients with radioresistance. Combining multiple clinicopathological characteristics, a decision tree and a nomogram were further built to respectively optimize the risk stratification for OS and to quantify risk assessment for individual patients. The E2F-related gene signature could provide a useful tool to distinguish high-risk LUSC patients with radioresistance who may benefit from adjuvant therapies, thus to facilitate personalized management.

## Data Availability Statement

Publicly available datasets were analyzed in this study. These data can be found here: “GSE42172,GSE29013,GSE30219,GSE37745,GSE14814, GSE17710, GSE42127, and GSE74777.” In addition, RNA-Seq data in FPKM of 499 patients who met the criteria were obtained from TCGA, and the expression data were taken as a validation set 2 after normalization by transcripts per kilobase per million (TPM).

## Author Contributions

CW conceived and designed the whole project and drafted the manuscript. XG and XZ analyzed the data and wrote the manuscript. MZ carried out data interpretations and helped data discussion. YC provided specialized expertise and collaboration in data analysis. All authors contributed to the article and approved the submitted version.

## Funding

This work was supported by the Key Project of Jiangsu Commission of Health (K2019030).

## Conflict of Interest

The authors declare that the research was conducted in the absence of any commercial or financial relationships that could be construed as a potential conflict of interest.

## Publisher’s Note

All claims expressed in this article are solely those of the authors and do not necessarily represent those of their affiliated organizations, or those of the publisher, the editors and the reviewers. Any product that may be evaluated in this article, or claim that may be made by its manufacturer, is not guaranteed or endorsed by the publisher.

## Supplementary Material

Supplementary Figure 1 Boxplot shows the transcriptional map before/after the removal of batch effects. **(A)** The transcriptional maps of three datasets before/after removing the batch effect training set. **(B)** The transcriptional maps of four datasets before/after removing the batch effect training set.

Supplementary Figure 2 Construction of a suitable megena network. **(A)** Diagnostic graph for each scale. **(B)** Seven was selected as the best scale, **(C)** Consensus matrix of 7 scales, **(D)**. Hub gene for each scale.

Supplementary Figure 3 A gene signature related to E2F is established. **(A)** MEGENA is performed with the whole transcriptome analysis data and the E2F ssGSEA Z score, and 70 modules were determined inseven scales. **(B)** Modules 25 and 71 were considered to have the closest correlation with E2F. **(C)** Fifty-three promising candidates were identified among the genes extracted from the 25 and 71 modules. **(D)** LASSO Cox regression model was utilized to identify the most reliable biomarker.

Supplementary Figure 4 A gene signature related to E2F is established. **(A)** The gene network of module 25 and its submodule 71. **(B)** LASSO Cox regression model was utilized to identify the most reliable biomarker.

Supplementary Figure 5 Verification of gene signatures in different sets. **(A**–**C)**. The subgroups of the three sets are assigned based on the optimum k value of the consensus cluster.
