# Supplementary figures and images for: Development and Validation of an E2F-Related Gene Signature to Predict Prognosis of Patients With Lung Squamous Cell Carcinoma
Source: Front Oncol. 2021 Oct 22;11:756096. doi: 10.3389/fonc.2021.756096 (PMC8569707; doi:10.3389/fonc.2021.756096)

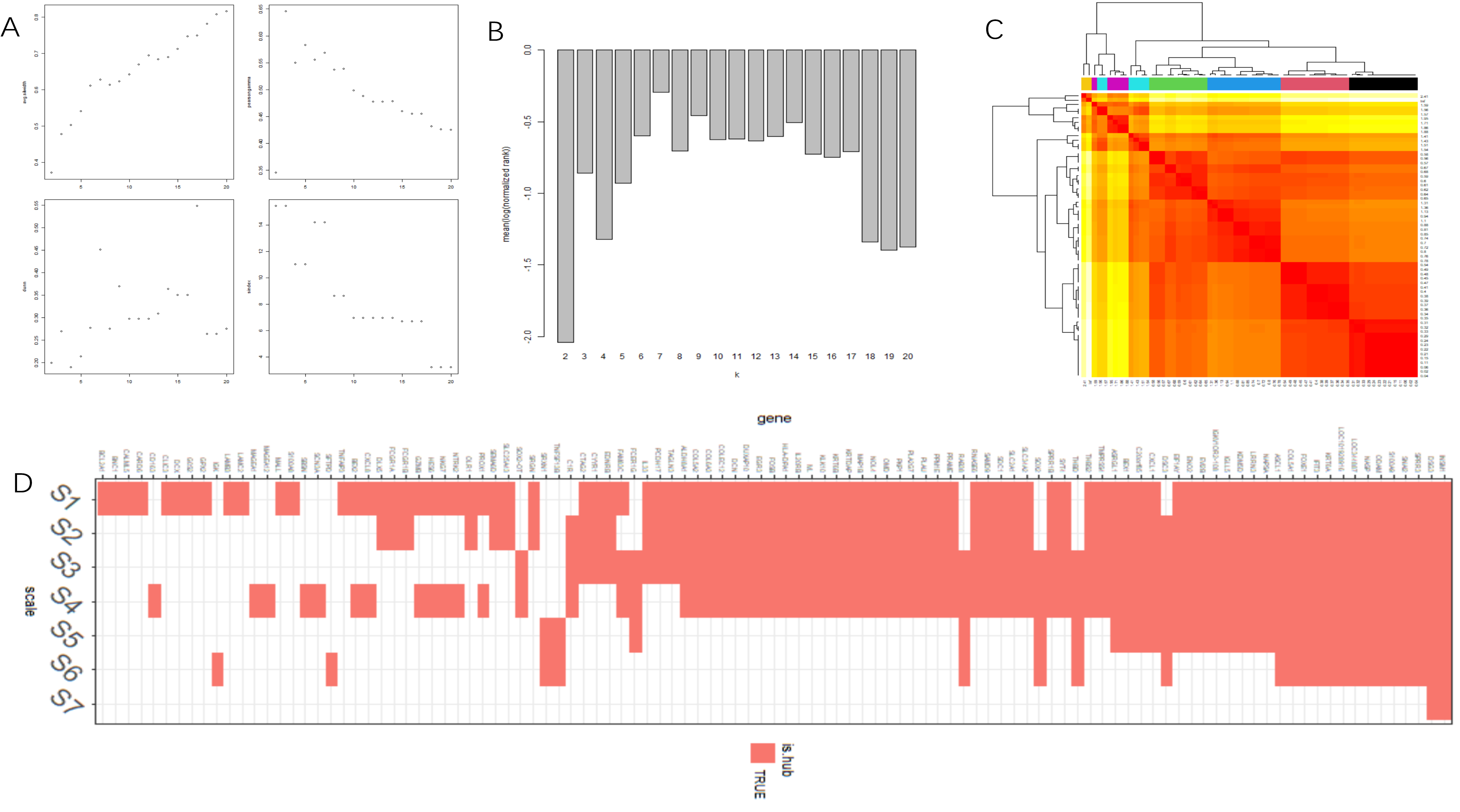

Supplement: Supplementary Figure 2 — Construction of a suitable megena network. (A) Diagnostic graph for each scale. (B) Seven was selected as the best scale, (C) Consensus matrix of 7 scales, (D). Hub gene for each scale. [file Image_2.tif]

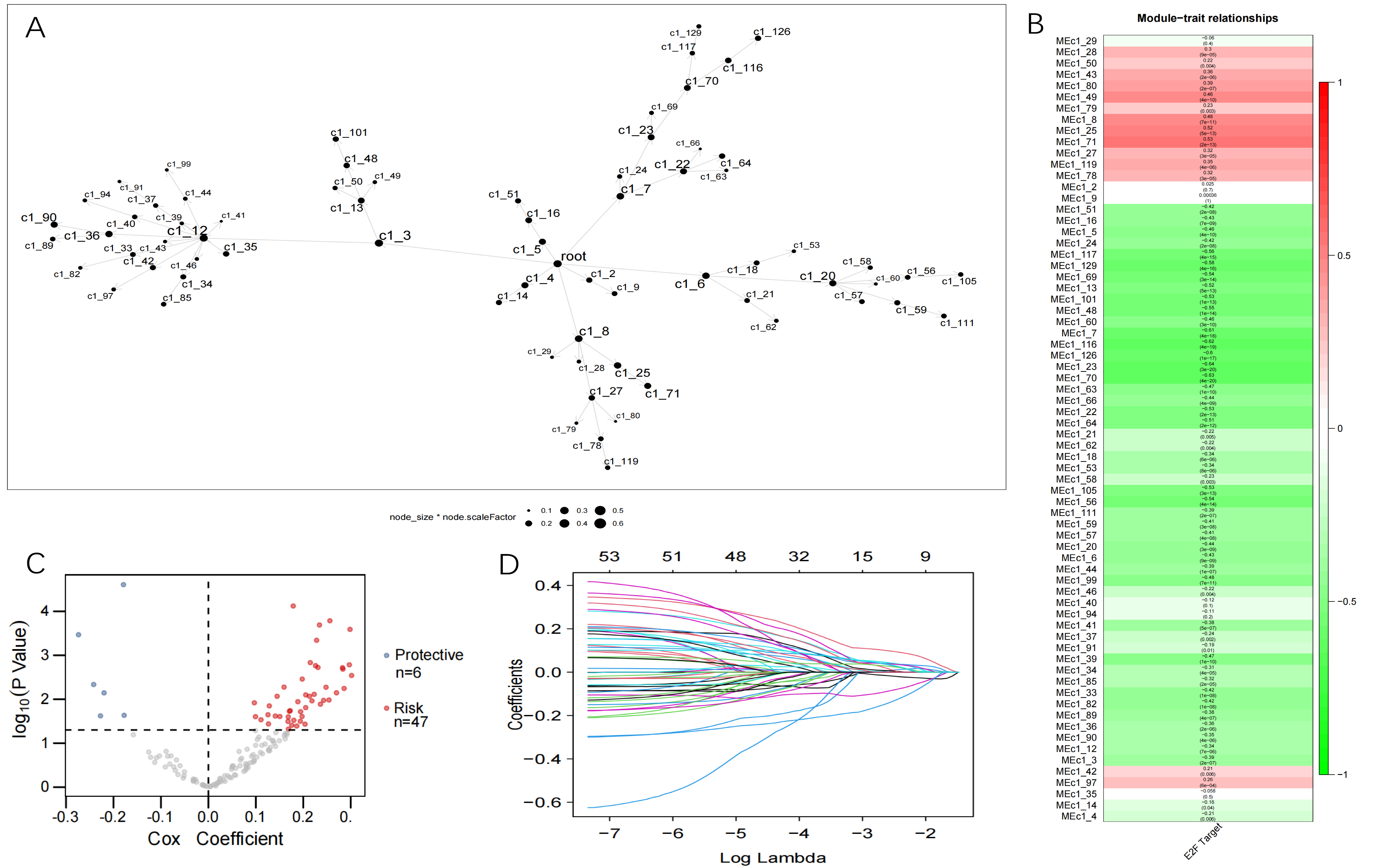

Supplement: Supplementary Figure 3 — A gene signature related to E2F is established. (A) MEGENA is performed with the whole transcriptome analysis data and the E2F ssGSEA Z score, and 70 modules were determined inseven scales. (B) Modules 25 and 71 were considered to have the closest correlation with E2F. (C) Fifty-three promising candidates were identified among the genes extracted from the 25 and 71 modules. (D) LASSO Cox regression model was utilized to identify the most reliable biomarker. [file Image_3.tif]

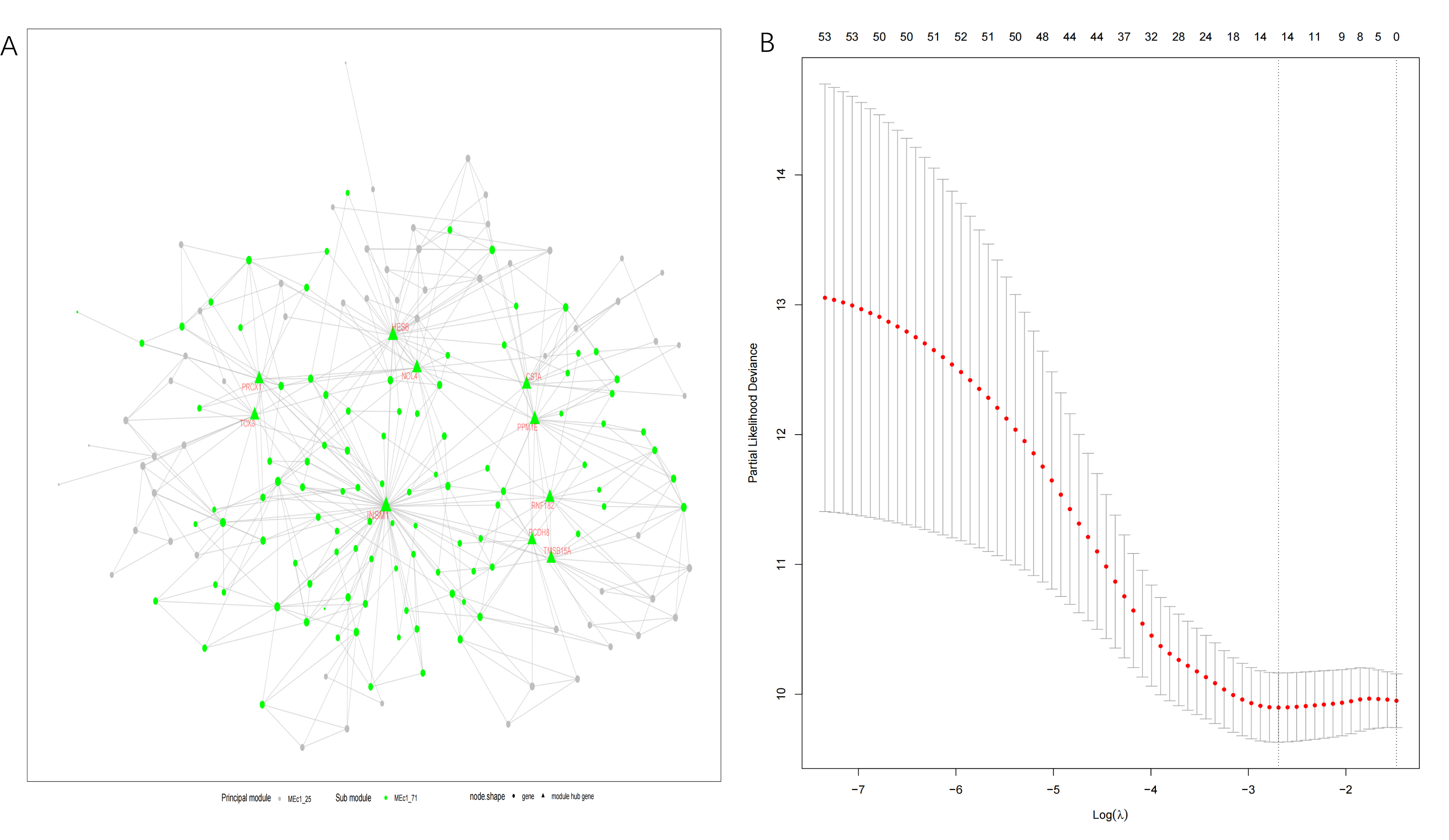

Supplement: Supplementary Figure 4 — A gene signature related to E2F is established. (A) The gene network of module 25 and its submodule 71. (B) LASSO Cox regression model was utilized to identify the most reliable biomarker. [file Image_4.tif]

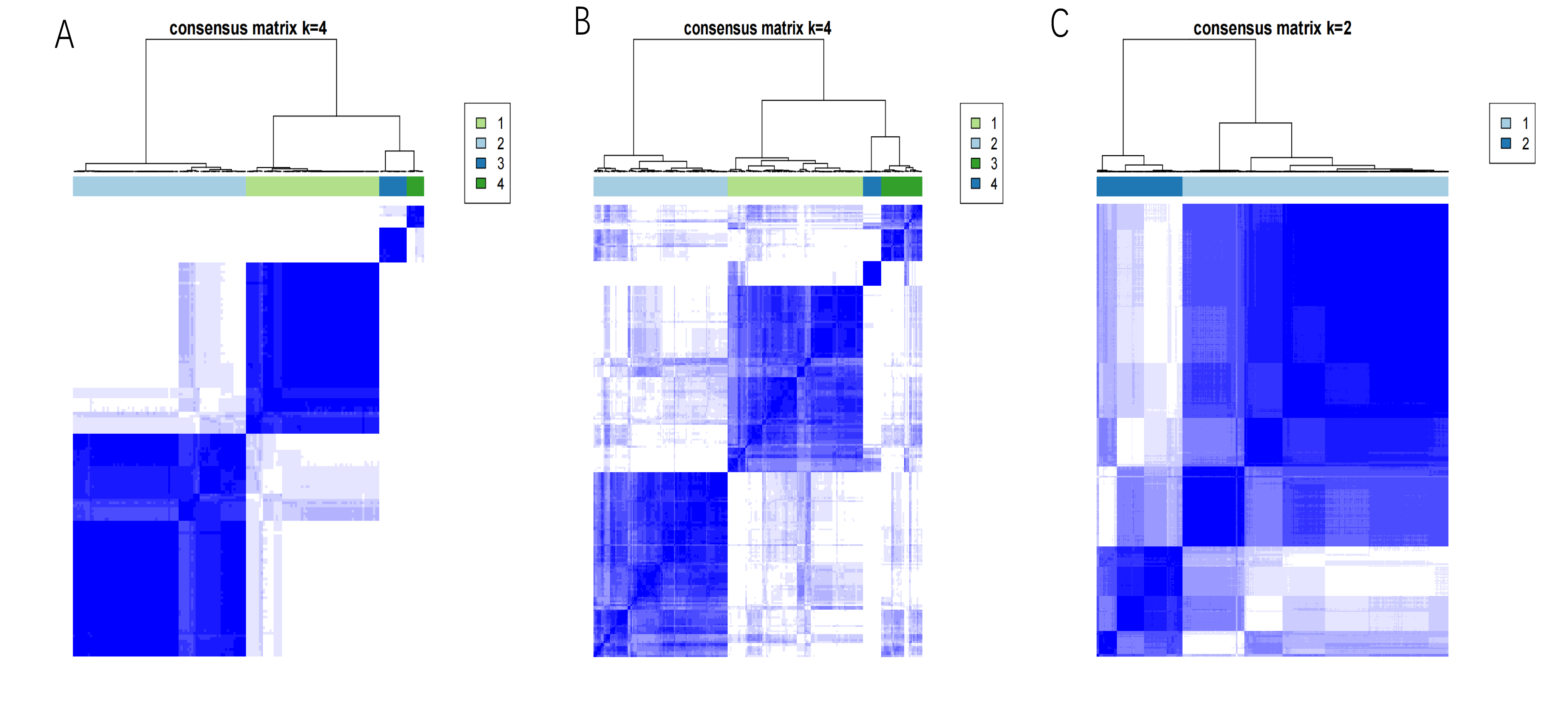

Supplement: Supplementary Figure 5 — Verification of gene signatures in different sets. (A–C). The subgroups of the three sets are assigned based on the optimum k value of the consensus cluster. [file Image_5.tif]
